# Contradiction between Plastid Gene Transcription and Function Due to Complex Posttranscriptional Splicing: An Exemplary Study of *ycf15* Function and Evolution in Angiosperms

**DOI:** 10.1371/journal.pone.0059620

**Published:** 2013-03-18

**Authors:** Chao Shi, Yuan Liu, Hui Huang, En-Hua Xia, Hai-Bin Zhang, Li-Zhi Gao

**Affiliations:** 1 Key Laboratory of Biodiversity and Biogeography, Kunming Institute of Botany, The Chinese Academy of Sciences, Kunming, China; 2 Plant Germplasm and Genomics Center, Germplasm Bank of Wild Species in Southwest China, Kunming Institute of Botany, The Chinese Academy of Sciences, Kunming, China; 3 University of the Chinese Academy of Sciences, Beijing, China; Kunming Institute of Zoology, Chinese Academy of Sciences, China

## Abstract

Plant chloroplast genes are usually co-transcribed while its posttranscriptional splicing is fairly complex and remains largely unsolved. On basis of sequencing the three complete *Camellia* (Theaceae) chloroplast genomes for the first time, we comprehensively analyzed the evolutionary patterns of *ycf15*, a plastid gene quite paradoxical in terms of its function and evolution, along the inferred angiosperm phylogeny. Although many species in separate lineages including the three species reported here contained an intact *ycf15* gene in their chloroplast genomes, the phylogenetic mixture of both intact and obviously disabled *ycf15* genes imply that they are all non-functional. Both intracellular gene transfer (IGT) and horizontal gene transfer (HGT) failed to explain such distributional anomalies. While, transcriptome analyses revealed that *ycf15* was transcribed as precursor polycistronic transcript which contained *ycf2*, *ycf15* and antisense *trnL-CAA*. The transcriptome assembly was surprisingly found to cover near the complete *Camellia* chloroplast genome. Many non-coding regions including pseudogenes were mapped by multiple transcripts, indicating the generality of pseudogene transcriptions. Our results suggest that plastid DNA posttranscriptional splicing may involve complex cleavage of non-functional genes.

## Introduction

Chloroplasts are semi-autonomous organelles that were derived from a cyanobacterial endosymbiont and entered the eukaryotic cell ancestor as an endosymbiont around one billion years ago [1], [2]. The subsequent coevolution of the chloroplast and nuclear genomes produced a highly compact chloroplast genome with its size significantly reduced to 120–200 kb [3]. However, in spite of its small size, the gene expression system of chloroplasts is far more complex than that of their cyanobacterial progenitor [4]–[6]. Usually, transcripts of higher plants chloroplast genomes undergo a variety of complex maturation events, including *cis*- and *trans*-splicing, cleavage of polycistronic messages, processing of 5′ and 3′ ends, and RNA editing [5]. In addition, plant chloroplast genomes contain many dispersedly distributed non-functional copies or pseudogenes fragments with numbers varied among different genomes, e.g. the *Pelargonium*×*hortorum* genome harbors at least 30 pseudogenes that account for ∼1/5 of its total genes [7]. It has long been considered that the most chloroplast functional genes are generally transcribed as single polycistronic transcripts with subsequently processed into smaller mature RNAs [5], [8]. Nevertheless, we still lack a genome-wide profile concerning the transcripton of these non-functional elements or pseudogenes in the chloroplast genome.

In this study, we initially focused on *ycf15*, a gene which has been attracted an intense attention during the past decade [7], [9]–[11]. The gene was first identified in *Nicotiana* chloroplast genome [12] with its expression similarly detected in Solanaceae chloroplasts by array hybridizations [13]. Since *ycf15* does not possess identifiable orthologues in eubacteria, it was presumed that the gene might descend from eukaryotic roots [9], [14] or originate from the lateral gene transfer [15], [16]. However, the validity of *ycf15* as a protein-coding gene has long been questioned [7], [10]. For example, it was disabled in some of the basal angiosperms such as *Amborella* [17] and *Nuphar* [11], monocots, most rosids, and some other separate lineages. And it has been wholly lost especially in some other lineages, e.g. *Illicium*, *Acorus*, *Ceratophyllum* and *Ranunculus* during evolution. Even so, its intact gene copy still remains in many species, e.g. several asterids, *Magnolia* [18] and *Piper* [19]. In addition, Schmitz-Linneweber and co-workers [9] also found that the plastid genomes of *Spinacia* and *Arabidopsis* contain *ycf15* as two pieces of the 5′ and 3′ sections are separated by 250–300 bp of ‘intervening sequence’, and the same situation was also present in many other species [11].

Although *ycf15* has been annotated in several sequenced chloroplast genomes, until now, it can almost not settle whether this gene is able to functionally encode a protein or how it has evolved in angiosperms so far. Since the ‘non-interleaved’ *ycf15* gene was only found in asterids, Raubeson *et al*. [11] assumed that, if it was really a protein-coding gene, it should be re-activated through the excision of the intervening sequence in asterids, and its pseudogenes should remain and be conserved in other lineages of angiosperms. To shed light on the function and evolution of *ycf15*, a systematic survey, including the distribution of both activated and inactivated forms among angiosperms, extra chloroplast genome sequences that contain intact *ycf15* gene sequences (e.g. plastid sequences) from asterids as well as patterns of gene expression, may be essential to unlock the mystery.

*Camellia* is an economically and phylogenetically important genus in family Theaceae [20]. Here, we first report the three complete chloroplast genome sequences of *Camellia*: *C. sinensis* var. *assamica*, *C. taliensis* and *C. oleifera.* They are, up to date, the only sequenced chloroplast genomes among Ericales, one lineage of asterids. Genome analyses showed that, among these three *Camellia* species, *ycf15* gene was intact and particularly comprised an ATG start codon in contrast to GTG discovered in other sequenced chloroplast genomes. Deep-sequencing of transcriptome of *C. sinensis* var. *assamica*, followed by transcriptome reads mapping, surprisingly uncovered an unexpected transcription profiling that a complete *C. sinensis* var. *assamica* chloroplast genome was nearly transcribed. Gene expression profiling of *ycf15* together with its extra transcriptional and evolutionary analyses showed that this gene may have involved a complex posttranscriptional splicing which may not be as simple as previously acknowledged. Considering that pseudogenes are ubiquitous in chloroplast genomes, the transcription of *ycf15* gene in this study thus point toward the complexity of the plant plastid RNA metabolisms.

## Results

### General Features of the Three *Camellia* Chloroplast Genomes and their Phylogenetic Relationships

**Figure 1:**
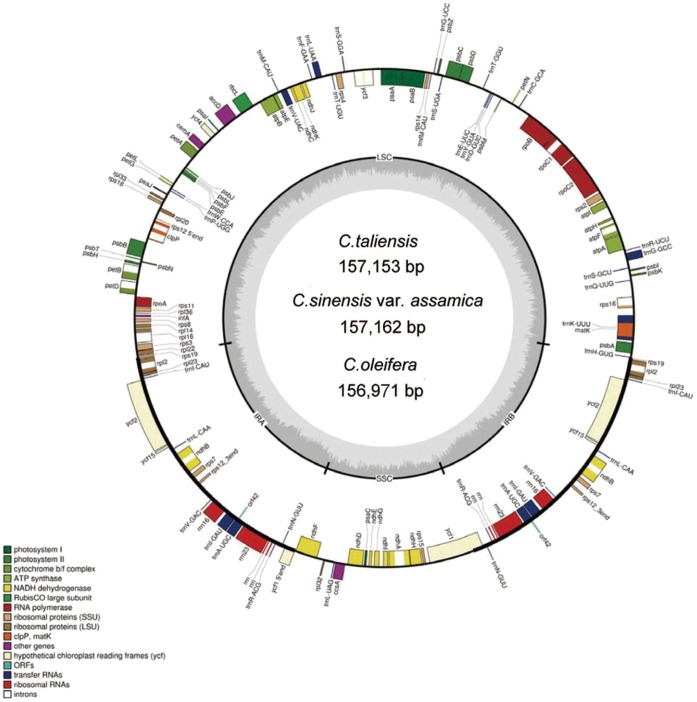
The map of the three *Camellia* chloroplast genome sequences. Genes on the outside of the map are transcribed in the clockwise direction and genes on the inside of the map are transcribed in the counterclockwise direction. Dashed area in the inner circle indicates the GC content of the chloroplast genome.

Sizes of the three determined camellia chloroplast genomes are 157,162 bp in *C. sinensis* var. *assamica,* 157,153 bp in *C. taliensis*, and 156,971 bp in *C. oleifera*, respectively (Table 1). All the three chloroplast genomes exhibited a typical quadripartite structure, consisting of a pair of inverted repeats (IRs) (26,071–26,134 bp) separated by a large single copy (LSC) (86,515–86,670 bp) and a small single copy (SSC) (18,285–18,341 bp) (Table 1). The three chloroplast genomes encoded an identical set of 133 genes with 19 of which were duplicated in the IR regions and 114 are unique (Figure 1). Among these unique genes, 15 included one intron and two have two introns. All of these coding regions account for 51.3% of the whole genome (Table 1). Genome-scale alignments revealed a high sequence similarity among these three species (99.6%) (Figure S1), and there were no obvious sequence inversions or rearrangement events. Seventy-five indels in all were found with sizes ranging from 1 to 90 bp (mean length, 6 bp; total length, 495 bp). The *C. oleifera* contained the two largest deletions (90 and 65 bp, respectively). Of all the detected indels, 71 (95%) were observed in intergenic sequences (IGS).

**Table 1:** Summary of the chloroplast genome sequencing, assembly and features.

	*C. sinensis* var. *assamica*	*C. taliensis*	*C. oleifera*
**Total no. of Illumina paired-end reads**	767,146	1,330,928	3,435,172
**Total no. of GS 20 reads**	36,427	72,664	18,948
**Mean coverage**	17.6	18.1	52.2
**Genome size (bp)**	157,162	157,153	156,971
a **LSC length (bp)**	86,609	86,670	86,515
b **SSC lengh (bp)**	18,285	18,341	18,288
c **IR length (bp)**	26,134	26,071	26,084
**Number of genes**	133	133	133
**Percent of coding regions**	51.3	51.3	51.3

a LSC, large single copy;

b SSC, small single copy;

c IR, inverted repeats.

**Figure 2:**
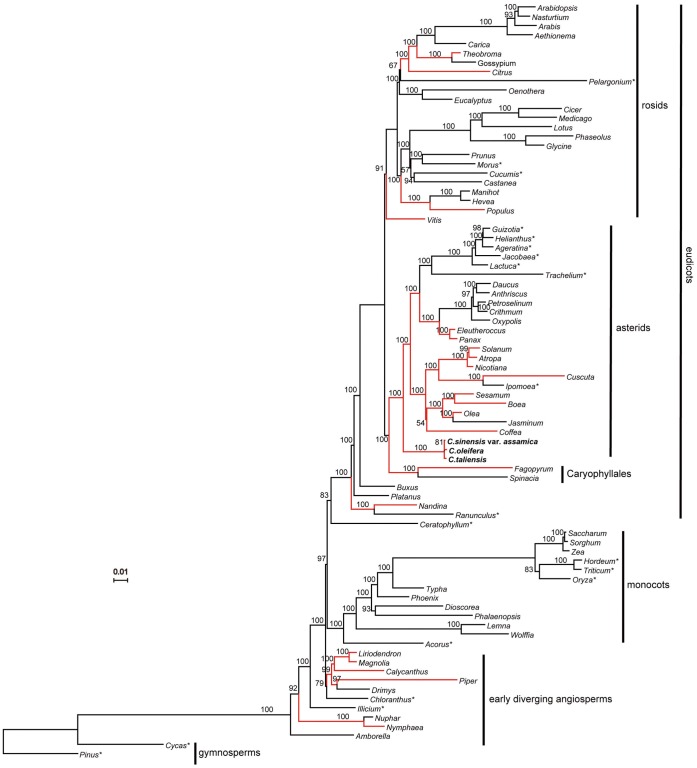
ML phylogram of the angiosperms. The tree has a –lnL = 938438.26. Numbers at the nodes are ML bootstrap support values. Three *Camellia* chloroplast genomes reported here are highlighted in bold. The red branches indicate that intact *ycf15* gene is present in related species and asterisks on the top right corner of the species indicate putative losses of *ycf15* gene. Scale bar indicates the increment of 0.01 substitutions per site.

To determine phylogenetic relationships of the three *Camellia* species with other major angiosperms clades, we employed 78 protein-coding genes from 82 taxa including 80 angiosperms and two outgroups of gymnosperms including *Pinus thunbergii* and *Cycas taitungensis*. Maximum likelihood (ML) analysis resulted in a single tree with –lnL = 938438.26 (Figure 2). Bootstrap analyses indicated that most of the nodes (64/76) were supported by values of 95% or greater and 58 of them had a bootstrap value of 100%. Maximum parsimony (MP) analysis also got similar topology (Figure S2). All major clades including basal angiosperms, monocots and eudicots were strongly supported, and the topology in this study was fairly similar to previous work by using plastid genome-scale data to resolve phylogenetic relationships among angiosperms [21]. The obtained phylogenies strongly supported that *Camellia* (Theaceae) were sister to the rest of the asterids with a bootstrap value of 100%, and the whole asterids were sister to Caryophyllales with a bootstrap value of 100%. However, phylogenetic relationships among the three *Camellia* species were not quite clearly resolved, as shown with low bootstrap supports and short branch lengths, indicating low levels of sequence divergence of their chloroplast protein-coding genes.

### Evolution of *ycf15* in Angiosperms

**Figure 3:**
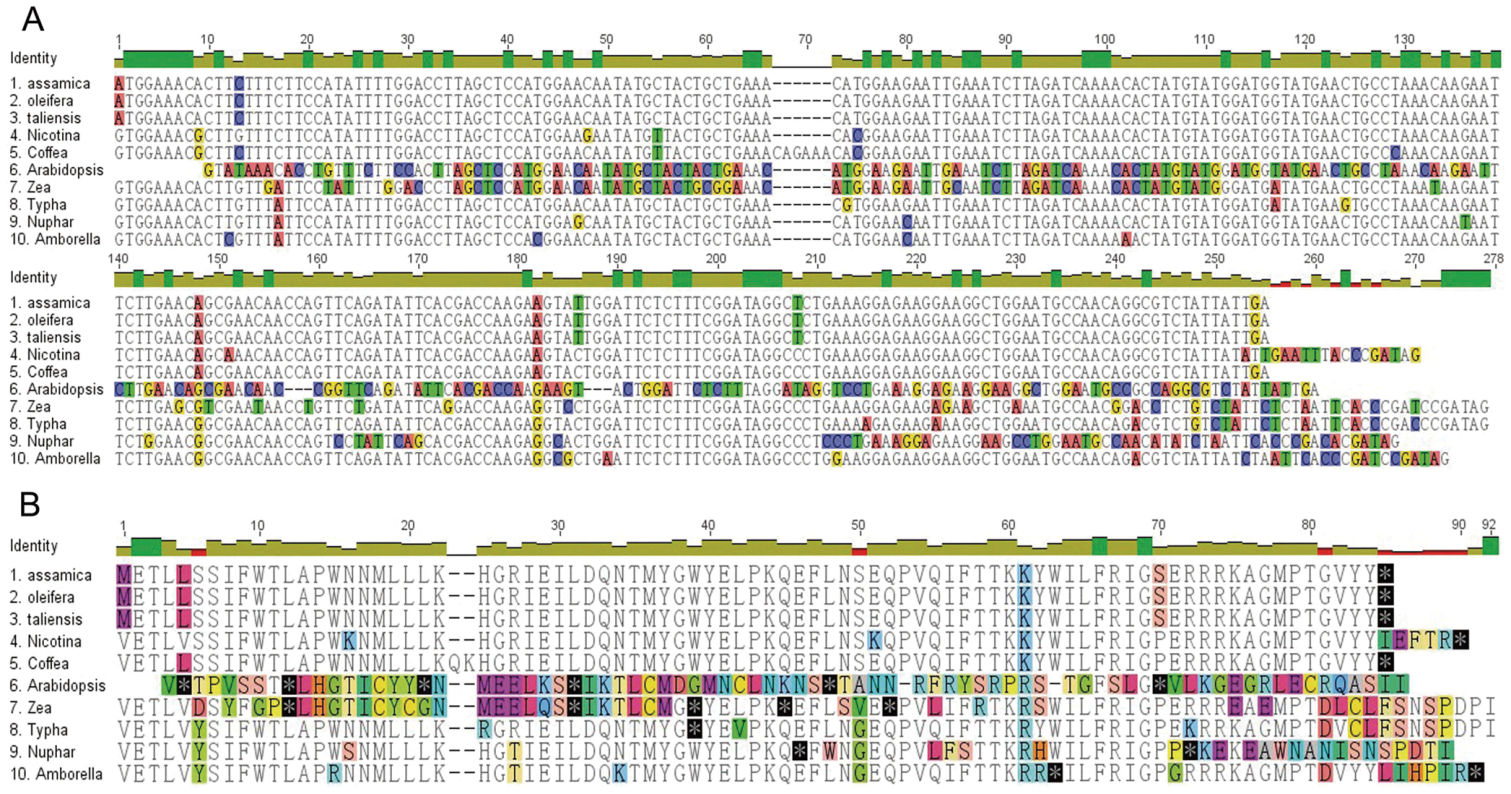
Alignment of the *ycf15* gene and protein sequences in the ten representative species of angiosperms. A) alignment of the *ycf15* gene sequences; B) alignment of the *ycf15* protein sequences. Black asterisks indicate stop codon in protein sequence.

*ycf15* sequences were retrieved after annotating of the three *Camellia* chloroplast genomes. Compared with those in other representative chloroplast genomes, *ycf15* in the *Camellia* employed an ATG start condon instead of GTG, which makes it more likely to be a functional gene in this genus. In addition, multiple internal stop codons were detected in many other species (as illustrated in Figure 3), suggesting that *ycf15* may be disabled in these species. To obtain a broad view on the evolution of *ycf15* across angiosperms, we further marked the distribution of this gene along the phylogenetic tree (Figure 2). The evolutionary patterns of y*cf15* genes showed that they discontinuously evolved with the species that contained an intact, obviously disabled, or lack orthologous sequences, which mixed together in the angiosperm phylogeny. In this study, *ycf15* was not observed in the examined chloroplast genome of gymnosperms and other species outside angiosperms; of ten species representing early diverging lineages of angiosperms, only five were found to contain an intact *ycf15*; in the next-diverging thirteen lineages of monocots under investigation, *ycf15* became either disabled or absent from the related species; in eudicots, we found that intact *ycf15* genes were mainly distributed at basal group of asterids together with some taxa of rosids.

**Figure 4:**
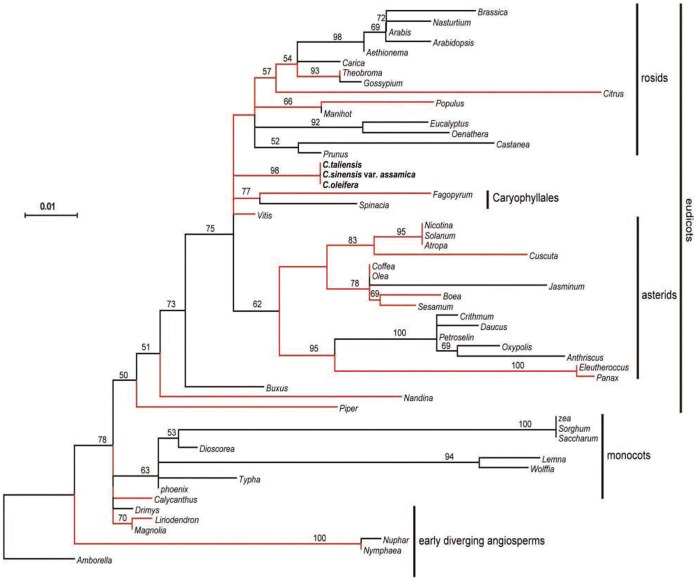
ML phylogram of 55 taxa based on *ycf15* gene sequences. The analyzed genes include both intact and disabled sequences. The tree has a –lnL = 1983.94. Bootstrap support values >50% are given at nodes. The red branches indicate that intact *ycf15* gene is present in related species. Scale bar indicates the increment of 0.01 substitutions per site.

Phylogenetic analysis of *ycf15* including both intact and non-functional gene copies were also performed among 55 taxa, while *ycf15* was totally lost from the other 27 species. The aligned data set contained 381 nucleotides. ML analysis generated a tree with –lnL = 1983.94 (Figure 4). Overall topology of this tree was similar to that inferred from 78 genes, although many nodes showed weak bootstrap supports lower than 50% (Figure 4).

### Transcriptome Reads Mapping and the Transcription Analyses of *ycf15*

**Figure 5:**
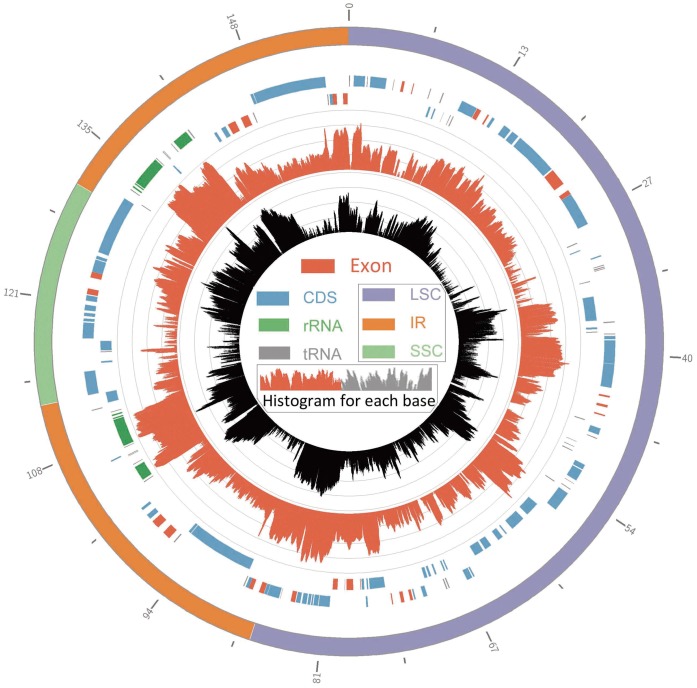
Circular plot of transcriptome reads mapping of the *C. sinensis* var. *assamica* chloroplast genome. The first ring shows genome structure of *C. sinensis* var. *assamica*, while the second and third rings show genes in the *Camellia* chloroplast genome that transcribed clockwise and counter clockwise, respectively. The next two rings show transcriptome reads from *C. sinensis* var. *assamica* and *C. sinensis* var. *sinensis* were separately mapped to the genome. Log_10_ of reads depth for each base was calculated and plotted across the genome. The figure was constructed using Circos [57].

To gain an in-depth insight into patterns of gene expression across the *Camellia* chloroplast genomes we analyzed the transcriptome data produced by RNA-Seq. The expression of chloroplast genes were first examined through chloroplast transcriptome data from *C. sinensis* var. *assamica*. We mapped all the raw sequence reads to *C. sinensis* var. *assamica* chloroplast genome to identify the pattern of gene expression. Intriguingly, up to 787,428 of the total reads were effortlessly mapped to the chloroplast genome of *C. sinensis* var. *assamica* with an average reads depth of 399×, although the failure to detect transcripts for one protein-coding gene, *petL*, and two tRNA genes, *trnT-UGU* and *trnT-GGU* (Supplementary Table S1). Moreover, the consensus sequence assembled from the mapped reads (152,566 bp long) covered 97.1% of the genome (Figure 5). Among all the genes, rRNAs displayed the most abundant transcripts, followed by genes encoding small/large subunit of ribosome and ATP synthetic genes. Multiple transcripts were attractively found to map to transcripts for many non-functional gene copies, for example, the pseudogenes of ψ*lhbA*, ψ*ycf68*, ψ*orf56* and ψ*orf 188* (they were not annotated in *Camellia* chloroplast genomes by reason of the presence of internal stop codons) and the truncated copies of *rps12*, *rps19* and *ycf1*, which were all nearly 100% covered by relevant reads. All the seven non-functional genes listed here were grouped into polycistronic transcription units that represent primary mRNAs of the plastid genome.

To further verify the above-described findings of transcriptome reads mapping, we collected transcriptome reads of *C. sinensis* var. *sinensis* [22] deposited in NCBI database and mapped them to the *C. sinensis* var. *assamica* chloroplast genome over again. The obtained 7,732,270 reads composed one-fifth of our obtained transcriptome sequences of *C. sinensis* var. *assamica*. There were 312,286 mapped to the chloroplast genome of *C. sinensis* var. *assamica*. A 144,602 bp consensus sequence was assembled; accounting for 92% of the genome with average reads depth of 133× (Figure 5). Accordingly, a nearly complete pseudo *C. sinensis* var. *sinensis* chloroplast genome was unexpectedly obtained by means of transcriptome data. Similar gene transcription information was also observed as in the transcriptome of *C. sinensis* var. *assamica* (Supplementary Table S1). Both two transcriptome datasets of *C. sinensis* apparently verified the observed transcripts of several non-functional genes, indicating that they are indeed transcribed (Supplementary Table S1).

Transcriptome mapping interestingly showed that multiple reads were mapped to *ycf15* (Figure S3 and Table S1). This finding thus indicated that *ycf15* was transcribed in the *Camellia* chloroplast genomes. The assembly of mapped reads demonstrated that *ycf15* was co-transcribed with the upstream *ycf2* as well as the downstream antisense *trnL-CAA*. It was further supported by BLAST searches against NCBI Transcriptome Shotgun Assembly (TSA) database using *ycf15* of *C. sinensis* var. *assamica* as a query sequence, where *ycf15* gene was aligned to the middle of the mRNA sequence (GenBank accession number: HP738268.1) of 2756 bp long from *C. sinensis* var. *sinensis* with a 100% sequence similarity. All together, our results provided evidences for that *ycf15* was indeed transcribed in its primary mRNA.

We further surveyed *ycf15* expression in other species through BLAST searches against the two NCBI databases of EST and TSA. Expression information of *ycf15* was found in two extra species, one was *Nicotiana tabacum* and the other was *A. trichopoda*. In the first case, *ycf15* was aligned to *N. tabacum* cDNA clone (GenBank accession number: AM808715.1) of 500 bp long with a 100% similarity; in the second case, *ycf15* was aligned to *A. trichopoda* cDNA clone (GenBank accession number: FD431960.1) of 656 bp long with a 100% similarity. The high similarities between *ycf15* and cDNA sequences suggested that the cDNA sequences did not experience posttranscriptional editing. Further speaking, it was indeed primary mRNA sequences of *ycf15* gene that were represented by cDNAs observed in the above-described cases of *N. tabacum* and *A. trichopoda*, showing that the GTG start codon in *ycf15* was not edited into standard ATG, since the G to U RNA editing in start codons of the chloroplast genomes was a common step for posttranscriptional editing [23]–[25]. Furthermore, a 294 bp ‘intervening sequence’ was not spliced out in cDNA sequence of *A. trichopoda*, and in fact, a stop codon was presented within the gene (Figure 3), indicating that it might be a pseudogene in this species. As a whole, results of our study showed that *ycf15* was firstly transcribed as precursor polycistronic transcript and the further posttranscriptional editing should be incorporated into pre-mRNA maturation.

### RNA Editing and Intron Splicing

RNA editing and intron splicing often act as major steps of chloroplast mRNA posttranscription. RNA editing in plastids of flowering plants changes hundreds of selected cytidines to uridines (C-to-U), mostly in coding regions of mRNAs. Reads-mapping of the transcriptome further identified a total of 31 complete or partial editing sites in the whole *C. sinensis* var. *assamica* chloroplast genome (Table 2). Among all the edited sites, 26 were found in coding regions that all caused an amino acid change (Table 2). In addition, of all the eleven protein-coding genes that contain one or two introns in *Camellia* chloroplast genomes, nine were detected to have intron splicing in our dataset (data not shown). Results presented here thus demonstrate that high-throughput transcriptome reads mapping may serve as a powerful method for the detection of RNA editing and intron splicing.

**Table 2:** RNA editing detected by transcriptome reads mapping.

Genes	Strands^a^	Position	Base^b^	A^c^	C^c^	G^c^	U^c^	Condon editing position^d^	Amino acid changes
***matK***	−	2314	G	21	0	1	0	**C**AT → **T**AT	H → Y
***matK***	−	2847	G	11	0	4	0	T**C**T → T**T**T	S → F
***matK***	−	3103	G	11	0	0	0	**C**AC → **T**AC	H → Y
***rps16*** ** intron**	−	5699	G	14	0	0	0	–	–
***psbK - psbI***	+	8518	C	0	28	0	29	–	–
***atpA***	−	11607	G	100	0	3	0	T**C**A → T**T**A	S → L
***atpA***	−	11730	G	196	0	4	0	C**C**A → C**T**A	P → L
***atpF***	−	13759	G	89	0	27	0	C**C**A → C**T**A	P → L
***rps2***	−	16993	G	231	0	2	0	T**C**A → T**T**A	S → L
***rps2***	−	17107	G	65	0	39	0	A**C**A → A**T**A	T → I
***rpoC2***	−	17896	G	178	0	24	0	T**C**A → T**T**A	S → L
***rpoC2***	−	18775	G	14	0	3	0	T**C**T → T**T**T	S → F
***rpoC1***	−	23350	G	9	0	5	0	T**C**A → T**T**A	S → L
***rpoC1***	−	24541	G	13	0	10	0	T**C**A → T**T**A	S → L
***rpoB***	−	25821	G	13	0	2	0	T**C**T → T**T**T	S → F
***psbZ***	+	37874	C	0	45	0	138	T**C**A → T**T**A	S → L
***rps14***	−	38898	G	346	0	50	0	C**C**A → C**T**A	P → L
***rps14***	−	38967	G	326	1	26	0	T**C**A → T**T**A	S → L
***ycf3*** ** intron**	−	45008	G	46	0	9	0	–	–
***trnL-UAA*** ** intron**	−	49664	G	0	0	16	60	–	–
***ndhK***	−	52696	G	8	0	4	0	T**C**A → T**T**A	S → L
***accD***	+	60548	C	0	9	0	30	C**C**T → C**T**T	P → L
***psaI***	+	61442	C	0	1	0	12	T**C**T → T**T**T	S → F
***psaI***	+	61447	C	0	3	0	7	**C**AT → **T**AT	H → Y
***psbF***	−	66717	G	88	0	15	21	T**C**T → T**T**T	S → F
***psbE***	−	66841	G	137	0	10	0	C**C**T → C**T**T	P → L
***trnW-CCA***	−	68908	G	16	0	0	0	–	–
***rps18***	+	70757	C	0	9	0	29	T**C**G → T**T**G	S → L
***petB***	+	78772	C	0	18	0	133	C**C**A → C**T**A	P → L
***ndhF***	−	114757	G	12	0	4	0	T**C**A → T**T**A	S → L
***ndhH***	−	125218	G	75	0	8	0	T**C**A → T**T**A	S → L

a Strands are indicated with “+”, positive strand, and “−”, negative strand;

b Base in the positive strand;

c Transcriptome reads that represent corresponding base substitutions that were counted;

d Underline indicates the edited base.

## Discussion

This study determined, for the first time, the three *Camellia* chloroplast genomes of Theaceae, and they are the only sequenced chloroplast genomes of Ericales up to the present time. All of the three genomes were found to possess a typical quartered genomic structure of angiosperms and exhibit a high interspecific conservation of the gene content and genomic arrangement. We interestingly observed that *ycf15*, a gene which has been paid great attention to its function by previous workers [7], [9]–[11], includes intact open reading frames (ORF) and ATG initial codons in all three *Camellia* genomes. The finding thus raised our interest to further investigate the evolution and function of *ycf15* in angiosperms and then explore the transcription of such non-functional genes in chloroplast genomes.

The phylogenetic tree constructed based on 78 protein-coding genes exhibited a clear evolutionary history of ycf15 in angiosperms. Overall, the distribution of the presumed ycf15 functional gene as an intact open reading frame was quite anomalous, since the mixture of both intact and disabled genes across the whole phylogenetic tree in angiosperms was different from evolutionary pattern observed in the other chloroplast genes. For example, many genes, including *infA* [26], *accD* [27], *ycf1*, and *ycf2* [28]–[30], were all present in the major lineages of early diverging angiosperms but lost from some lineages of the later diverging clades. The evolution of *ycf15* was left us more confused about its anomalous distribution in angiosperms.

Three biological models came to mind to account for the observed anomaly in this study. The first is that functional gene of *ycf15* in these species that lack an intact *ycf15* copy in the chloroplast genome may be transferred to the nucleus [14], [31], the second alternative is that functional copy of *ycf15* in the chloroplast genome was from the nucleus of the same plant through intracellular gene transfer (IGT) [15], and the third is that the re-acquisition of functional gene copies of *ycf15* may be the result of horizontal gene transfer from unrelated lineage of plants (HGT), a biological mechanism that was found in both mitochondrial [15] and chloroplast genomes [16]. However, our further analyses appeared not to support all three hypotheses. Firstly, BLAST searches failed to detect a functional gene copy in the nuclear genomes of neither the species that lacks an intact gene copy of *ycf15* nor the one that possesses an intact *ycf15* gene copy in their chloroplast genomes, indicating that IGT has never occurred in these species. Secondly, a phylogenetic tree was constructed by using *ycf15* gene sequences to examine whether HGT was involved in *ycf15* gene evolution. The topology of *ycf15* gene tree was largely consistent with the phylogeny of angiosperms with the exception that some lineages were not well separated with relatively low bootstrap values. Thus, the hypothesis that HGT was involved in the evolution of *ycf15* may be rejected, as it could be expected that intact sequences of *ycf15* come from different lineages should be phylogenetically clustered together with enough high bootstrap supports [15].

Previous studies suggest that gene transfer from the plastid to nucleus occurred quite often during the plastid evolution [32]–[34]. Indeed, it has reasonably explained some events of gene loss (e.g. *infA*, *accD*, *rpl22*) during angiosperm evolution. In addition, HGT was used to account for phylogenetic anomalies of some mitochondrial genes [15], and recently, HGT was also observed between chloroplast genomes [16]. The failure of all three mechanisms to explain this phenomenon suggests that *ycf15* should not be a functional gene in angiosperms.

Transcriptome sequencing is able to supply sufficient information for gene expression and functional studies nowadays. By the convenience of transcriptome sequencing, we analyzed patterns of gene expression in C. sinensis var. assamica. It was found that almost all of the annotated chloroplast genes were expressed. Interestingly, transcripts of ycf15 genes were also detected in Camellia and other two lineages of Nicotiana and Amborella. Further analyses suggest that ycf15 was co-transcribed with ycf2 and antisense trnL-CAA, a phenomenon which has never been observed before [3], [35].

This finding brings about one major question: it has been claimed that ycf15 is a pseudogene, why its transcripts could still be detected in the sequenced transcriptome? Our results showed that these transcripts represent both primary and processed mRNA sequences of the plastid genome. That is, *ycf15* gene sequence should be removed from the pre-mRNA after transcription termination in order to activate the function of other genes. In the whole chloroplast transcriptome mapping, raw transcriptome sequence reads covered more than 97% of the Camellia chloroplast genome, including the amount of non-functional gene copies and IGS sequences, indicating that many non-functional sequences were transcribed and retained in plastid primary mRNA. In addition, earlier studies based on Northern hybridization analyses reported that more than 90% of the chloroplast genome was transcribed in the pea and rice [36], [37]; recent progress in understanding how chloroplast precursor RNA processes and stabilizes further showed that the regulation of plastid gene expression mainly occurred at posttranscriptional stage 3840. In combination with results in the present study, it can be presumed that chloroplast transcripts represent a population of mRNAs probably generated by primary RNA processing [39].

Chloroplast genes with related or unrelated functions can be all co-transcribed together [8], [35], [41], while chimeric gene transcripts that include nonfunctional gene copy or pseudogene were rarely observed. Although the chloroplast genomes are quite conserved in their gene order and content, some taxa still remain a highly rearranged chloroplast genome which contains many nonfunctional gene copies or pseudogenes [7], [42]. It thus can be expected that the transcription of these genomes are quite complex as well [4], [43]. Our finding also suggests the limitation by using high-throughout RNA-Seq to detect functional transcripts of the chloroplast genes, leading to the observation of unexpected expression profiling of many non-functional gene copies.

## Methods

### Chloroplast Genome Sequencing

Fresh leaves from the two *Camellia* species of *C. sinensis* var. *assamica* and *C. taliensis* were harvested from Yunnan Tea Tree Research Institute, Menghai, Yunnan Province, China, while fresh leaves of *C. oleifera* were collected from plants grown at Kunming Botanical Garden of Kunming Institute of Botany, Chinese Academy of Sciences. Voucher herbarium specimens were deposited at the Herbarium of Kunming Institute of Botany (KUN). For each species, the purified chloroplast DNA (cpDNA) was isolated from about 20 g fresh leaves with an improved high salt method as reported formerly [44].

After DNA isolation, DNA libraries were subsequently constructed and sequenced by following Illumina (Solexa) and Roche (454) sequencing protocols. Sequence reads of 2×100 bp and an average length of 300 bp were obtained for Illumina (Solexa) and Roche (454), respectively. For genome assembly, three steps were performed to *de novo* assemble the chloroplast genomes. First, the filtered Illumina (Solexa) and Roche (454) reads were assembled into contigs using SOAPdenovo (Li *et al*., 2010) and Newbler (Roche), respectively, then these contigs from two different assembly sources were combined using Amos sofware package, an Open-Source whole genome assembler (http://sourceforge.net/projects/amos/files/). Second, contigs were aligned to the reference chloroplast genome of *Coffea arabica* using Blat program [45], as a result, the aligned contigs were ordered according to the position of the reference genome. Third, the assembled draft genomes were again mapped with raw reads and visualized in Geneious (version 5.1) [46], in which most gaps can be replaced with raw reads mapped to draft genome. To check the accuracy of sequence assembly, regions with ambiguous read mapping (conflicted reads mapped to the same genomic region) and low coverage (≤2 reads) were verified by PCR amplifications and Sanger sequencing. Here, 4 to 27 primer pairs (Supplementary Table S2) were used to close the gaps and verify sequence assembly.

### Genome Annotation

Annotation of the sequenced genomes was performed through DOGMA [47] using default parameters to predict protein-coding genes, transfer RNA (tRNA) genes, and ribosome RNA (rRNA) genes. Start and stop codons of protein-coding genes were searched and determined by BLASTX against the NCBI protein database. The gene maps of the three genomes were drawn by using OGDraw (version 1.2) [48]. Genome sequence data from this article can be found in the EMBL/GenBank data libraries under accession number JQ975030–JQ975032.

### Phylogenetic Tree Construction

For the purpose of phylogenetic analyses, a total of 78 protein-coding genes were collected from the 82 species of angiosperms, representing major plant lineages for which chloroplast genomes were sequenced (see Supplementary Table S3 for a complete list of the chosen genomes). For each species, gene sequences were extracted from the plastid genome, translated into amino acid sequences, and aligned in MSWAT (http://mswat.ccbb.utexas.edu/). After manual adjustment, this alignment was used to constrain the nucleotide alignment again. Some genes (e.g. *infA*, *rpl22*, and *accD*) that got lost from the majority of species were excluded from the subsequent data analyses. Maximum likelihood (ML) analyses were performed on RaxML version 7.2.6 [49] with GTR nucleotide substitution model and 1,000 bootstrap replicates. For the construction of *ycf15* phylogenetic tree, 55 sequences were separately extracted from plant chloroplast genomes that contain the nearly complete *ycf15*. If an ‘intervening sequence’ was present in *ycf15*, it was then removed before further data analyses. All sequences were aligned, codon by codon, using MEGA5 [50] and ML tree was constructed with the same criterion.

### RNA Isolation and Transcriptome Sequencing

Total RNA was extracted from mature leaves of *C. sinensis* var. *assamica* using standard phenol/chloroform RNA isolation method, and then was treated with DNase I for 30 min at 37°C (New England BioLabs) to remove residual DNA. Isolated RNA was fragmented by the RNA fragmentation kit (Ambion); first strand cDNA was synthesized using random hexamer-primer and reverse transcriptase (Invitrogen). The second-strand cDNA was synthesized using RNase H (Invitrogen) and DNA polymerase I (New England BioLabs).

For the high-throughput sequencing, the sequencing library was constructed by following the manufacturer’s instructions (Illumina). Fragments of ∼300 bp were excised and enriched by PCR for 18 cycles. The products were loaded onto flow cell channels at a concentration of 2 pM for paired-end 100 bp×2 sequencing. The Illumina GA processing pipeline (version 0.2.2.6) was applied for image analysis and base calling. A total of 36,663,434 raw reads were obtained after the sequencing.

### Transcriptome Reads Mapping

Raw Illumina reads were obtained after base calling in the Solexa Pipeline (version 0.2.2.6). We removed the adapters and trimmed the low quality bases using SolexaQA package (parameters: -h 20 -b; -l 30) [51]. The filtered RNA-seq reads (Phred quality scores >20, length >30) were then mapped to the *C. sinensis* var. *assamica* chloroplast genome using Bowtie (parameters: –best, -S, default options otherwise) [52]. Using the SAMtools package [53], alignment results was indexed as BAM files. Coverage and base depth were done by converting the BAM alignments into pileup files.

### Computational Identification of RNA Editing Sites and Intron Splicing Events

In order to identify the RNA editing sites, all short reads of transcriptome from *C. sinensis* var. *assamica* were again mapped to its chloroplast genome using PASS software (version 1.62) [54]. The uniquely mapped reads with size ≥30 bp and Phred quality scores >20 were reserved (parameters: -flc 1, -fid 90, -fle 30, -gff, -info gff, -trim 5 20). The reads mapping results (GFF file) were then used to identify C-to-U changes due to RNA editing in the *C. sinensis* var. *assamica* chloroplast genome by the pass snp program (parameters: -f 0.5 -q 20 -c 10 2000). Briefly, pass_snp program takes the alignment file (GFF file) as input, then finds putative RNA editing sites, checking quality, coverage and frequency for each base transition [55]. A site is considered potentially edited if reads depth ≥10 and five or more U in the aligned reads at the same position. To detect intron splicing events from protein-coding genes containing introns of the *C. sinensis* var. *assamica* chloroplast genome, GTF/GFF3 input file of the genome sequence without annotation information was used to detect the spliced reads called as “junctions”. The reads of transcriptome from *C. sinensis* var. *assamica* were then blasted against its chloroplast genome by using TopHat with default parameters [56].

### Transcriptome Data of *C. sinensis* var. *sinensis*

To verify our transcriptome reads mapping of *C. sinensis* var. *assamica*, we downloaded transcriptome reads of *C. sinensis* var. *sinensis* sequenced by Shi *et al*. [22] from the National Center for Biotechnology Information (NCBI) Short Read Archive with the accession number SRX020193. This sequence data includes 7,732,270 raw reads with mean length of 75 bp and was obtained from a pooled cDNA library of seven tissues, including tender shoots, young leaves, mature leaves, stems, young roots, flower buds and immature seeds. These raw reads were also trimmed using SolexaQA [51] with the same parameters as described above. Since *C. sinensis* var. *sinensis* chloroplast genome was not sequenced which may share a large sequence similarity with *C. sinensis* var. *assamica,* we mapped sequence reads to *C. sinensis* var. *assamica* chloroplast genome by using Bowtie (version 0.12.3) [52] with the same settings as described above.

## Supporting Information

Figure S1 **Visualization of alignments among the three** ***Camellia*** **species,** ***Coffea*** **,** ***Olea*** **, and** ***Amborella*** **chloroplast genome sequences.** VISTA-based identity plots show sequence identity among the six sequenced chloroplast genomes with *C. sinensis* var. *assamica* as a reference. Genome regions are color-coded as coding and noncoding regions.
(PDF)

Figure S2 **MP phylogram of the angiosperms.** Numbers at the nodes are MP bootstrap support values.
(PDF)

Figure S3 **Transcriptome reads mapping of** ***ycf15*** **and its flanking sequences.** This is a screenshot as a part of transcriptome mapping to the whole chloroplast genome. Transcriptiome reads are shown as yellow bars and these reads cover both up- and down-streams of *ycf15*.
(PDF)

Table S1 **Transcriptome reads mapping of the** ***Camellia sinensis*** **var.** ***assamica*** **chloroplast genome.**
(PDF)

Table S2 **Primers used for validating genome assembly and gaps closing.**
(PDF)

Table S3 **Taxa included in the phylogenetic analyses with GenBank accession numbers.**
(PDF)
