# Supplementary figures and images for: Contradiction between Plastid Gene Transcription and Function Due to Complex Posttranscriptional Splicing: An Exemplary Study of ycf15 Function and Evolution in Angiosperms
Source: PLoS One. 2013 Mar 18;8(3):e59620. doi: 10.1371/journal.pone.0059620 (PMC3601113; doi:10.1371/journal.pone.0059620)

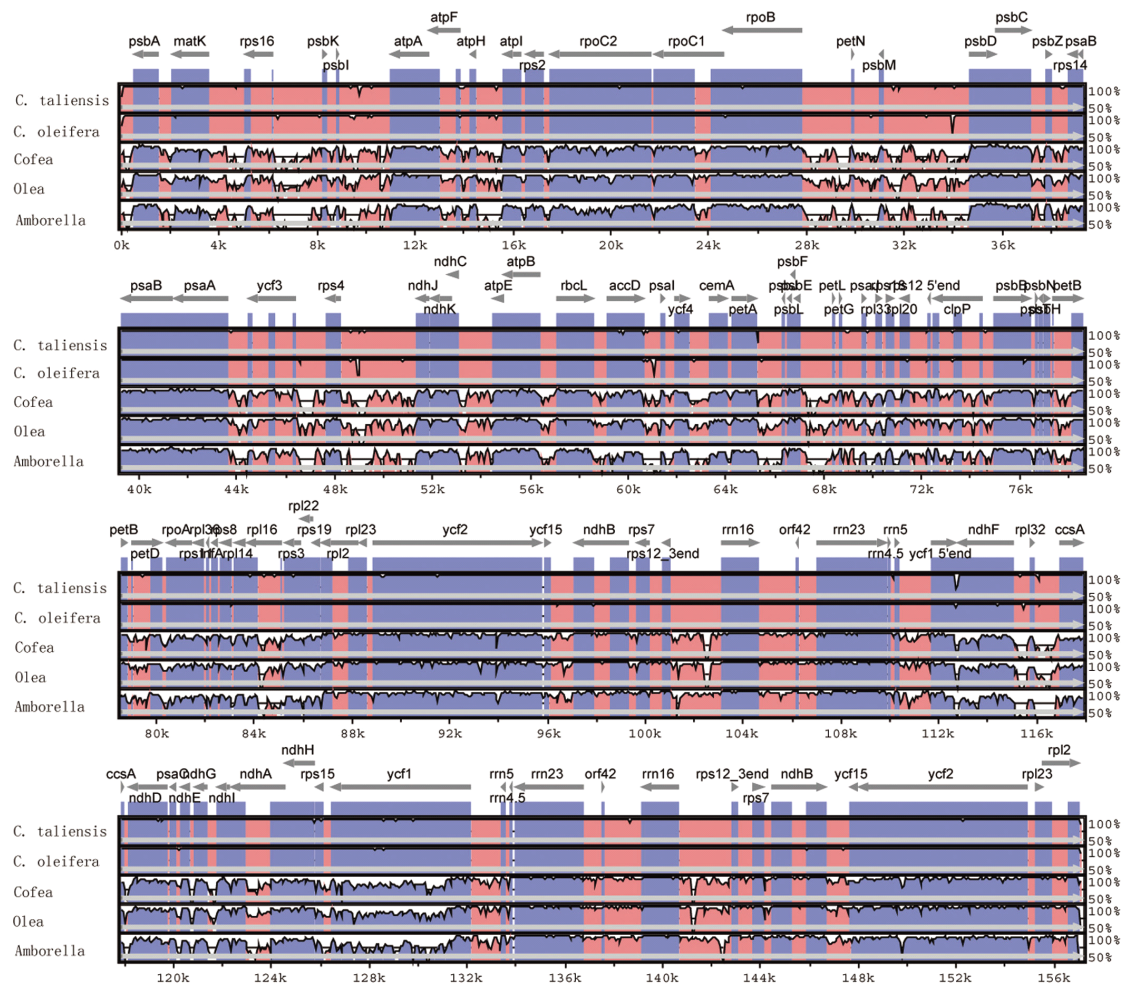

Supplement: Figure S1 — Visualization of alignments among the three Camellia species, Coffea , Olea , and Amborella chloroplast genome sequences. VISTA-based identity plots show sequence identity among the six sequenced chloroplast genomes with C. sinensis var. assamica as a reference. Genome regions are color-coded as coding and noncoding regions. (PDF) [file pone.0059620.s001.pdf]

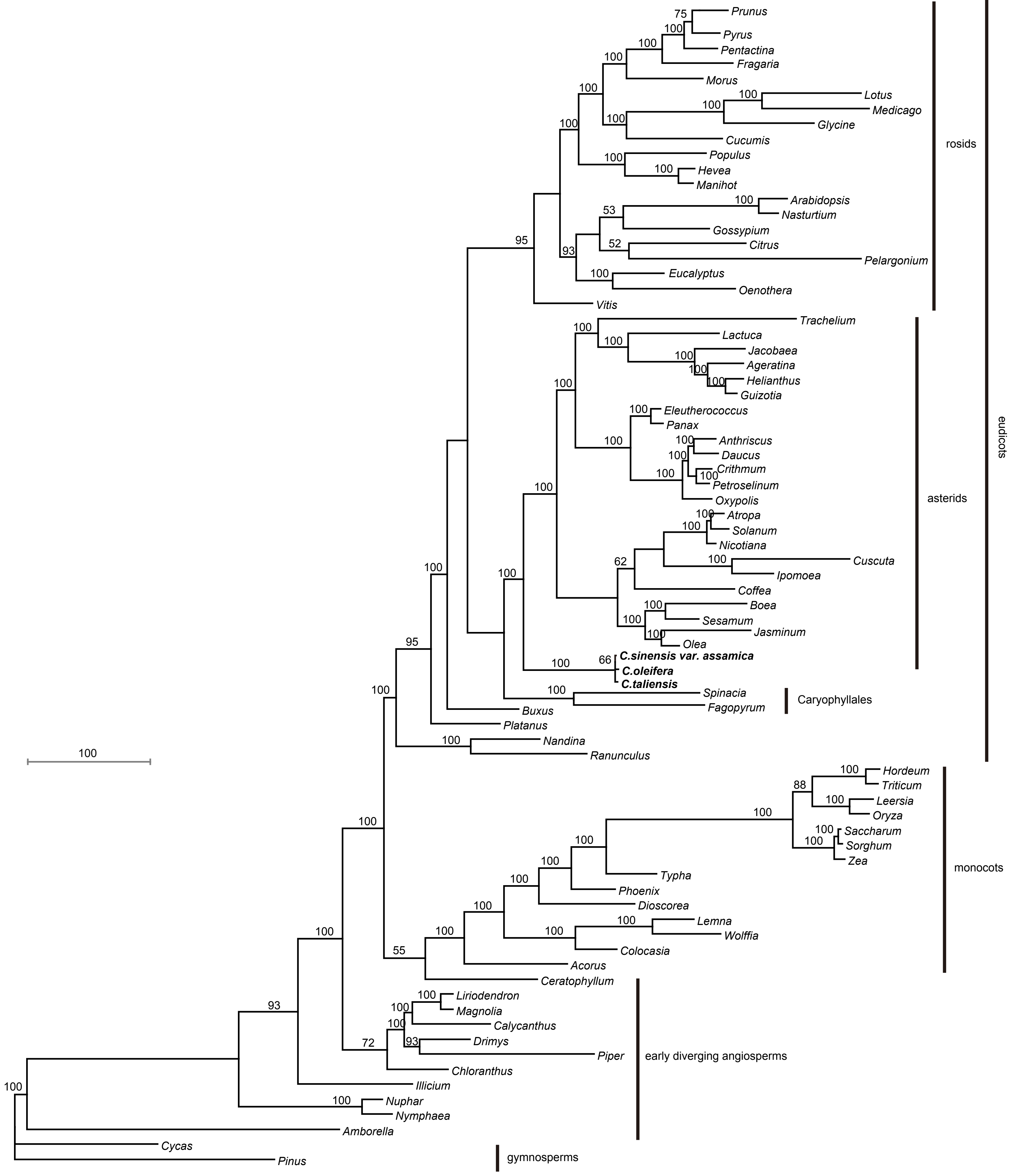

Supplement: Figure S2 — MP phylogram of the angiosperms. Numbers at the nodes are MP bootstrap support values. (PDF) [file pone.0059620.s002.pdf]

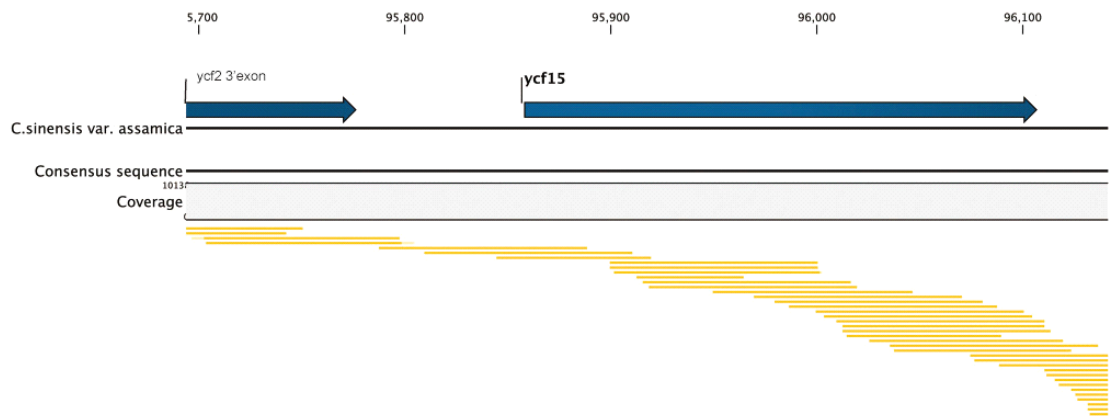

Supplement: Figure S3 — Transcriptome reads mapping of ycf15 and its flanking sequences. This is a screenshot as a part of transcriptome mapping to the whole chloroplast genome. Transcriptiome reads are shown as yellow bars and these reads cover both up- and down-streams of ycf15. (PDF) [file pone.0059620.s003.pdf]
