# Supplementary material for: Contradiction between Plastid Gene Transcription and Function Due to Complex Posttranscriptional Splicing: An Exemplary Study of ycf15 Function and Evolution in Angiosperms
Source: PLoS One. 2013 Mar 18;8(3):e59620. doi: 10.1371/journal.pone.0059620 (PMC3601113; doi:10.1371/journal.pone.0059620)
Supplement: Table S1 — Transcriptome reads mapping of the Camellia sinensis var. assamica chloroplast genome. (PDF) [file pone.0059620.s004.pdf]

Table S1 –Transcriptome reads mapping of the *Camellia sinensis* var. *assamica* chloroplast genome.

Gene name	Strand	Start position (bp)	End position (bp)	Gene size (bp)	Reads number	Coverage[a]	Reads numb er	Coverage[b]
trnH-GUG	-	3	77	74	12	78.67%	70	100%
psbA	-	463	1521	1058	5984	100%	329	100%
trnK-UUU	-	1742	1776	34	14	100%	3	100%
matK	-	2048	3547	1499	426	100%	86	70%
trnK-UUU	-	4264	4300	36	35	100%	22	100%
rps16	-	5056	6178	1122	275	100%	315	100%
trnQ-UUG	-	7793	7864	71	4	100%	5	100%
psbK	+	8198	8383	185	127	100%	65	100%
psbI	+	8780	8890	110	32	100%	21	100%
trnS-GCU	-	9033	9120	87	47	100%	94	100%
trnG-GCC	+	9796	9818	22	13	100%	18	100%
trnG-GCC	+	10524	10560	36	11	100%	28	100%
trnR-UCU	+	10812	10883	71	15	100%	25	100%
atpA	-	10997	12520	1523	1625	100%	590	100%
atpF	-	12580	13850	1270	951	100%	1578	100%
atpH	-	14227	14472	245	686	100%	353	100%
atpI	-	15577	16320	743	674	100%	1115	100%
rps2	-	16530	17240	710	1808	100%	196	100%
rpoC2	-	17487	21611	4124	1634	100%	359	88%
rpoC1	-	21789	24581	2792	356	100%	65	92.62%
rpoB	-	24608	27820	3212	521	100%	65	63.52%
trnC-GCA	+	29056	29136	80	8	100%	11	100%
petN	+	29846	29935	89	8	100%	0	0%
psbM	-	31018	31122	104	58	100%	99	100%
trnD-GUC	-	31950	32023	73	12	100%	3	45.95%
trnY-GUA	-	32136	32219	83	15	100%	6	100%
trnE-UUC	-	32279	32351	72	4	91.78%	4	97.26%
trnT-GGU	+	33125	33196	71	0	0%	20	100%
psbD	+	34715	35776	1061	1898	100%	4040	100%
psbC	+	35724	37145	1421	3580	100%	3549	100%
trnS-UGA	-	37378	37469	91	106	100%	340	100%
psbZ	+	37825	38013	188	306	100%	880	100%
trnG-UCC	+	38285	38355	70	37	100%	69	100%
trnfM-CAU	-	38517	38590	73	10	100%	129	100%
rps14	-	38744	39046	302	869	100%	2711	100%
psaB	-	39170	41374	2204	11265	100%	366	100%

psaA	-	41400	43652	2252	11377	100%	358	99.91%
ycf3	-	44441	46404	1963	592	100%	4028	100%
trnS-GGA	+	47253	47339	86	31	100%	8	96.55%
rps4	-	47632	48237	605	44	100%	26	78.38%
trnT-UGU	-	48571	48643	72	0	0%	14	100%
trnL-UAA	+	49624	49660	36	18	100%	382	100%
trnL-UAA	+	50184	50233	49	12	100%	38	100%
trnF-GAA	+	50606	50678	72	10	100%	3	97.26%
ndhJ	-	51346	51822	476	105	100%	92	100%
ndhK	-	51928	52776	848	222	100%	402	100%
ndhC	-	52656	53018	362	74	100%	204	100%
trnV-UAC	-	53374	54035	74	23	100.00%	88	100%
trnM-CAU	+	54202	54273	71	6	100%	152	100%
atpE	-	54477	54878	401	1991	100%	5293	100%
atpB	-	54875	56371	1496	14998	100%	17960	100%
rbcL	+	57124	58551	1427	17530	100%	773	100%
accD	+	59131	60621	1490	570	100%	234	100%
psaI	+	61363	61473	110	17	100%	7	100%
ycf4	+	61894	62448	554	319	100%	38	100%
cemA	+	63348	64043	695	129	100%	96	100%
petA	+	64283	65245	962	464	100%	148	95.12%
psbJ	-	66282	66404	122	137	100%	72	100%
psbL	-	66535	66651	116	175	100%	27	100%
psbF	-	66674	66793	119	249	100%	30	100%
psbE	-	66803	67054	251	488	100%	51	100%
petL	+	68347	68442	95	1	2.08%	1	50%
petG	+	68625	68738	113	39	100%	54	100%
trnW-CCA	-	68862	68935	73	19	100%	22	100%
trnP-UGG	-	69106	69179	73	56	100%	21	100%
psaJ	+	69570	69704	134	167	100%	469	100%
rpl33	+	70159	70359	200	33	100%	107	100%
rps18	+	70537	70842	305	73	100%	72	100%
rpl20	-	71105	71458	353	51	100%	137	100%
ψ***rps12***	-	72239	72352	113	28	100%	40	100%
clpP	-	72502	74482	1980	173	100%	283	100%
psbB	+	74935	76461	1526	7894	100%	382	100%
psbT	+	76643	76750	107	65	100%	13	100%
psbN	-	76806	76937	131	67	100%	44	100%
psbH	+	77040	77261	221	184	100%	75	100%
petB	+	77385	78809	1424	1320	100%	345	97.33%
petD	+	78998	80228	1230	631	100%	162	100%
rpoA	-	80434	81441	1007	4706	100%	2177	100%
rps11	-	81507	81923	416	9897	100%	2365	100%
rpl36	-	82038	82151	113	1245	100%	810	100%

infA	-	82268	82501	233	6019	100%	1808	100%
rps8	-	82623	83030	407	6316	100%	5543	100%
rpl14	-	83220	83588	368	9435	100%	3791	100%
rpl16	-	83701	85130	1429	15240	100%	8782	100%
rps3	-	85259	85915	656	1611	100%	2349	100%
rpl22	-	85900	86367	467	1048	100%	1567	100%
rps19	-	86419	86697	278	671	100%	1605	100%
rpl2	-	86758	88252	1494	13889	100%	7762	100%
rpl23	-	88271	88552	281	1613	100%	1115	100%
trnI-CAU	-	88718	88791	73	44	100%	5	96.19%
ycf2	+	88880	95776	6896	3231	100%	462	87.77%
ycf15	+	95857	96105	248	84	100%	22	97.57%
trnL-CAA	-	96476	96556	80	9	100%	6	98.83%
ndhB	-	97113	99324	2211	1292	100%	5815	100%
rps7	-	99650	100117	467	1808	100%	9621	100%
ψrps12	-	100713	100964	251	2425	100%	9699	100%
trnV-GAC	+	102809	102880	71	12	100%	4	72.78%
rrn16	+	103108	104597	1489	187198	100%	10356	100%
trnI-GAU	+	104894	104935	75	4071	100%	188	100%
trnA-UGC	+	105981	106018	37	2511	100%	116	100%
orf42	-	106148	106276	128	5685	100%	207	100%
trnA-UGC	+	106831	106865	34	2203	100%	44	100%
rrn23	+	107018	109826	2808	438212	100%	78277	100%
rrn4.5	+	109925	110027	102	2653	100%	401	100%
rrn5	+	110248	110368	120	112	100%	50	100%
trnR-ACG	+	110622	110695	73	30	100%	117	100%
trnN-GUU	-	111291	111362	71	64	100%	142	100%
ψycf1	+	111675	112730	1055	672	100%	1922	100%
ndhF	-	112800	115046	2246	352	100%	326	79.88%
rpl32	+	115726	115887	161	33	100%	21	100%
trnL-UAG	+	116809	116888	79	404	100%	205	100%
ccsA	+	116978	117943	965	32	77.85%	70	83.44%
ndhD	-	118182	119696	1514	47	88.58%	47	66.07%
psaC	-	119825	120070	245	90	100%	72	100%
ndhE	-	120301	120606	305	18	90.52%	50	100%
ndhG	-	120841	121371	530	31	100%	61	100%
ndhI	-	121766	122269	503	108	100%	330	100%
ndhA	-	122364	124539	2175	307	100%	4389	100%
ndhH	-	124541	125722	1181	997	100%	2062	100%
rps15	-	125815	126087	272	210	100%	715	100%
ycf1	-	126470	132097	5627	4112	96.94%	22890	100%

[a] Transcriptome reads were from *C. sinensis* var. assamica and [b] *C. Sinensis* var. *sinensis.*
