# Supplementary material for: Contradiction between Plastid Gene Transcription and Function Due to Complex Posttranscriptional Splicing: An Exemplary Study of ycf15 Function and Evolution in Angiosperms
Source: PLoS One. 2013 Mar 18;8(3):e59620. doi: 10.1371/journal.pone.0059620 (PMC3601113; doi:10.1371/journal.pone.0059620)
Supplement: Table S2 — Primers used for validating genome assembly and gaps closing. (PDF) [file pone.0059620.s005.pdf]

**Table S2 – Primers used for validating genome assembly and gaps closing.**

| *C. sinensis var. assamica* | | | |
|---|---|---|---|
| **primer** | **forward** | **reverse** | **position** |
| **as1** | CACTGCCTTGATCCACTT G | CAT TAA CCG TGC TAA CCT TG | 46 - 562 |
| **as2** | CGA *ACC* CAT CAT TTA CCT | CAT CTA TCC CAA TGA GCC | 4517 - 5294 |
| **as3** | CCA AGA GCA CCT TCA TTC C | GGA ACA TAG AGC ACA AGG AT | 6065 - 7584 |
| **as4** | AAC GAG ACG GAA GGA TTT | GCT TTG GCT TTA CTC ACG | 14765 - 115910 |
| **as5** | CGG ATC GTA TTA CTA CAG AGT C | TTG ATA CCA CCA GGA ATA GG | 18317 - 19388 |
| **as6** | TAT TCT CCG TAT CAG GTA TTT | CGA TGA CTT ACG CCT TAC C | 32887 - 33176 |
| **as7** | AAA GTA AGT GGA CCT GAC CC | GGT AAA TCC ACG AAC TCA AT | 33501 - 34550 |
| **as8** | TAT CAA CCA CTC AGC CAT CT | AAT CCT CCA AGC TAC CAA C | 37448 - 37800 |
| **as9** | ACC CGT TGT ATT TGC TTC TC | AAA TCC TGT CTC CGC AAC | 37892 - 38536 |
| **as10** | TAG GCA TAG GTA GCA ATC CAT | GCT ACG CCT TGA ACC ACT | 46418 - 47284 |
| **as11** | AGG *ACC* AGA AGT AGC AGG AT | AAT CCC TCC CTA CAA CTC AT | 56341 - 57122 |
| **as12** | TAG GTA GGG ATG ACA GGA TT | TTC ATT CCT TCC CGT GT | 69102 - 69730 |
| **as13** | ACT ATG ATG GTT CCG TTG C | TTAC CCT AAT CAA CCG ACT T | 72977 - 73629 |
| **as14** | ATT TAG GCA GAA TAC CGT CA | CTG GAG GGA TAA GAG GAT T | 112381 - 113010 |
| **as15** | AGT TCT GCT TCG GTA TTG C | TGG ATG GGA ATG AAT GAA G | 117230 - 127643 |
| **as16** | GAT TGT GCT TGT TCT ATC AGT | GGG AAT GGA GGC TGA ACT | 128912 - 129330 |
| **as17** | TTC CAT AAC TTT GCC GTG T | CTA AAC AGG AAC AAG AGG GA | 130884 - 131285 |
| **as18** | CAC TTG GCT *ACC* CAG CGT TT | GCA CCT GGA CAG AAA GAC CCT | 134008 - 134695 |
| **as19** | CGG TAG TAC CCT CGT TGA CTT | CAA CAG TCG GAC AAG TGG G | 86105 - 87020 |
| **as20** | CTT CGT CGC CGT AGT AAA TAG | GGC TCC CTA TTC AGT GCT ATG | 156995 - 901 |

| *C. taliensis* | | | |
|---|---|---|---|
| **primer** | **forward** | **reverse** | **position** |
| **ta1** | TGGGAGCAATGAATAGGTACGAAT | AAGACCGCTCAATAAATGAAATGCC | 4940 - 5542 |
| **ta2** | CGTTGCTTTCTACCACATCGTTTC | CAGTCTATCTGATAAATCCCCATTC | 6138 - 6825 |
| **ta3** | TTAGCAATCCGCCGCTTTAGTCC | AAAAGGACTTTGTTTCTACCGAGCT | 9082 - 10203 |
| **ta4** | ATGGATTCGCTGTTTGCTTACTGGC | CCCCAGATGGTGAAAGAATCGTCGT | 25078 - 25567 |
| **ta5** | GTAGACATTCCTTCATTTCCATCCC | ACCATTAACTATTGACTACGGACTG | 27785 - 28761 |
| **ta6** | CATATTGCCAACGAATTTACAGTCC | TTGAACCGATGACTTACGCCTTACC | 32166 - 33157 |
| **ta7** | CCGACAATGTAATGGAGAATGGAT | TCCTTTATTGGTCTATAACCCTGAC | 33959 - 34578 |
| **ta8** | AAAATAGTTGGAATCGCCCTGAAGA | TTTGGTAGCTCGCAAGGCTCATAAC | 38167 - 38550 |
| **ta9** | TAATCATTGAGTCCTCCTCTTTCCG | CTTAGTAATGGCTAATGACTGCTTG | 43646 - 44077 |
| **ta10** | TTCTGAGTGAGTTATTTCAGCTCCAC | GCCGCATGTTATACCATATTTCACT | 60560 - 61545 |
| **ta11** | GCTACAGCAGCCGGTATAGTAAGC | CCGTTCTATTTCCCGCTTATT | 64789 - 65580 |
| **ta12** | CCCTCTGGTTGAATCATAACGACTT | GACTAGAAGGAATCGGCGGAGAAAT | 82310 - 82642 |
| **ta13** | TCCCTTCATTCTTCCTCTATGTTGT | TAGTGATTCATTGGTCGGGATGG | 84054 - 84570 |
| **ta14** | GGCATTACGACCTTTACCACAACGA | TTTTCCGATCCAGTTCCTCCACCAC | 88123 - 89417 |
| **ta15** | AGCAACAACATAATGGAGGCAGTC | AAATGGTGATATTGTTCCTCCGTTG | 91005 - 91742 |
| **ta16** | GGAGCAAAAGGTATGATCCACG | GAATTCCTACCCCAGAACTAGAAAT | 115003 - 116140 |

| | | | |
|---|---|---|---|
| **ta17** | CTGGGAAGAACCAAAGTTTCAGATT | TATGGATCACCATTCCAAGT | 127345 - 127798 |
| **ta18** | AATACATATGCTTGTGACAAGGAGG | TCTGACTGATAGAACAAGCACAATC | 128482 - 128910 |
| **ta19** | AAATGGAATGGTATCCCAGGCTTCT | AAGGTTCAATGCGAGCCCAAAGATG | 129690 - 129970 |
| **ta20** | TGCCGTATCCAGACTAATACCAATC | ATCTAGCATTGGGTAGACCTCATAC | 131507 - 131823 |
| **ta21** | TGGTCTGTGAAGATGCGTTGTTAGG | TCCAAGGCACATTAGCATGGCGTAC | 138467 - 138740 |
| **ta22** | CCCAAGGACTCAATCGTATGGATAT | GACGTGACATGAGCGTGAAAGAGGT | 143364 - 143640 |
| **ta23** | AATGGTGATATTGTTCCTCCGTTG | GAGCAGAAGAGCCGTTTTCAAGTAG | 152010 - 152371 |
| **ta24** | TTGTAGGTATAATGGTGGAT | AAGCGTCCTGTAGTAAGAG | 85782 - 86940 |
| **ta25** | TCCGAGTGAATGGAAAGG | AAGGCATCAAATATGTAGGG | 112584 - 112890 |
| **ta26** | ATAAACTGGGTGGAAACG | AAACAGGAACAAGAGGGA | 130849 - 131284 |
| **ta27** | AGTTATGAACCCTGTAGACC | TAAGGCAGTGGATTGTGA | 156851 - 52 |

| ***C. oleifera*** | | | |
|---|---|---|---|
| **primer** | **forward** | **reverse** | **position** |
| **ol1** | TAACATTAGCGACGAGAT | TATGAACCCTGTAGACCA | 86208 - 86710 |
| **ol2** | CGAAACTACCTGTTATCC | TAGGAGCGGGACTTCTTC | 114226 - 114770 |
| **ol3** | TTTTATGTATCGCAACGG | AAACAGGAACAAGAGGGA | 130731 - 131058 |
| **ol4** | AAGCGTTGGCTAGGTAAG | TCAGGAAGGCGTTATTGT | 156597 - 389 |
