# Supplementary material for: Contradiction between Plastid Gene Transcription and Function Due to Complex Posttranscriptional Splicing: An Exemplary Study of ycf15 Function and Evolution in Angiosperms
Source: PLoS One. 2013 Mar 18;8(3):e59620. doi: 10.1371/journal.pone.0059620 (PMC3601113; doi:10.1371/journal.pone.0059620)
Supplement: Table S3 — Taxa included in the phylogenetic analyses with GenBank accession numbers. (PDF) [file pone.0059620.s006.pdf]

Table S3 – Taxa included in the phylogenetic analyses with GenBank accession numbers.

Classification	Taxon	Genbank accessions
**Gymnosperms**		
	Pinus thunbergii	NC_001631
	Cycas taitungensis	NC_009618
**Early diverging groups**		
	Amborella trichopoda	NC_005086
	Nymphaea alba	NC_006050
	Nuphar advena	NC_008788
	Illicium oligandrum	NC_009600
	Chloranthus spicatus	NC_009598
	Drimys granadensis	NC_008456
	Piper cenocladum	NC_008457
	Calycanthus floridus var. *glaucus*	NC_004993
	Magnolia kwangsiensis	NC_015892
	Liriodendron tulipifera	NC_008326
	Nandina domestica	NC_008336
	Ranunculus macranthus	NC_008796
	Platanus occidentalis	NC_008335
	Buxus microphylla	NC_009599
	Ceratophyllum demersum	NC_009962
**Monocots**		
	Acorus calamus	NC_007407
	Wolffia australiana	NC_015899
	Lemna minor	NC_010109
	Phalaenopsis aphrodite	NC_007499
	Dioscorea elephantipes	NC_009601
	Phoenix dactylifera	NC_013991
	Typha latifolia	NC_013823
	Oryza nivara	NC_005973
	Triticum aestivum	NC_002762
	Hordeum vulgare	NC_008590
	Zea mays	NC_001666
	Sorghum bicolor	NC_008602
	Saccharum hybrid cultivar SP-80-3280	NC_005878
**Rosids**		
	Vitis vinifera	NC_007957
	Populus alba	NC_008235
	Hevea brasiliensis	NC_015308
	Manihot esculenta	NC_010433

	Castanea mollissima	NC_014674
	Cucumis sativus	NC_007144
	Prunus persica	NC_014697
	Morus indica	NC_008359
	Glycine max	NC_007942
	Phaseolus vulgaris	NC_009259
	Lotus japonicus	NC_002694
	Cicer arietinum	NC_011163
	Medicago truncatula	NC_003119
	Cicer arietinum	NC_011163
	Eucalyptus grandis	NC_014570
	Oenothera argillicola	NC_010358
	Pelargonium x hortorum	NC_008454
	Citrus sinensis	NC_008334
	Gossypium arboreum	NC_016712
	Theobroma cacao	NC_014676
	Carica papaya	NC_010323
	Aethionema cordifolium	NC_009265
	Arabis hirsuta	NC_009268
	Nasturtium officinale	NC_009275
	Arabidopsis thaliana	NC_000932
**Caryophyllales**		
	Spinacia oleracea	NC_002202
	Fagopyrum esculentum	NC_010776
**Asterids**		
	Camellia sinensis* var. *assamica	**JQ975030**
	C. oleifera	**JQ975031**
	C. taliensis	**JQ975032**
	Coffea arabica	NC_008535
	Jasminum nudiflorum	NC_008407
	Olea europaea	NC_013707
	Boea hygrometrica	NC_016468
	Sesamum indicum	NC_016433
	Ipomoea purpurea	NC_009808
	Cuscuta exaltata	NC_009963
	Nicotiana tabacum	NC_001879
	Atropa belladonna	NC_004561
	Solanum bulbocastanum	NC_007943
	Panax ginseng	NC_006290
	Eleutherococcus senticosus	NC_016430
	Oxypolis greenmanii	NC_015832
	Crithmum maritimum	NC_015804
	Petroselinum crispum	NC_015821
	Anthriscus cerefolium	NC_015113

	Daucus carota	NC_008325
	Trachelium caeruleum	NC_010442
	Lactuca sativa	NC_007578
	Jacobaea vulgaris	NC_015543
	Ageratina adenophora	NC_015621
	Helianthus annuus	NC_007977
	Guizotia abyssinica	NC_010601
